# Seizing the Teachable Moment: A Pilot Study of Preventive Lifestyle Education in the Emergency Department

**DOI:** 10.7759/cureus.74825

**Published:** 2024-11-30

**Authors:** Daniel C Keyes, Dylan L Arroyo, Ghadah W Abdulshafi, Batoul Dabajeh, Joshua Polgar, Rima Sakr, Sakibur Hasan

**Affiliations:** 1 Emergency Medicine, Trinity Health Livonia Hospital, Livonia, USA; 2 Medicine, University of Michigan Medical School, Ann Arbor, USA; 3 Research, University of Michigan - Dearborn, Dearborn, USA; 4 Research, Western Michigan University Homer Stryker M.D. School of Medicine, Kalamazoo, USA; 5 Biostatistics, Michigan State University College of Osteopathic Medicine, East Lansing, USA

**Keywords:** education, emergency medicine, lifestyle medicine, patient education, prevention, transtheoretical model of change

## Abstract

Background

Preventive measures are critical in avoiding and limiting the severity of diseases. Key lifestyle behaviors include sleep hygiene, habitual exercise, a healthy diet, and avoidance of risky substances, particularly the use of tobacco. The transtheoretical model (TTM) of change suggests that patients can move towards healthful changes through education. This emergency department (ED) pilot study assessed patients’ readiness and confidence to adopt healthy lifestyle behaviors after receiving a brief video educational intervention. The study also investigated the impact of this preventive education on patient satisfaction.

Methods

Adult patients with low-acuity chest pain were prospectively randomized to a 10-minute video-based educational intervention. The sample size of 105 patients was chosen to demonstrate feasibility and provide data that could be used to design a larger study with a more diverse population. The outcome of this study used validated Readiness-to-Change and Confidence-to-Change questionnaires, along with selected questions from the U.S. HCAHPS (Hospital Consumer Assessment of Healthcare Providers and Systems) Patient Satisfaction Survey. A Student t-test was used to measure the difference in means between comparison groups where normal distribution could be assumed. A logistic regression model assessed the relationship between various factors and participants’ readiness in changing their lifestyle behaviors.

Results

Of the 105 subjects enrolled in the study, 63 were female, and 42 were male. This pilot study found a nonsignificant increase in Readiness-to-Change among those older (40-80 years) who watched the video, more than those younger (18-39 years). This study shows positive trends toward patients being receptive to change and increased patient satisfaction when an educational video is shown. Feasibility was demonstrated by using a video prevention tool in the ED.

Conclusion

The ED presents a unique “teachable moment” for preventive lifestyle health education. However, lifestyle behavioral health interventions have been largely absent from this setting. This pilot study demonstrates the feasibility of a video teaching model for use while patients wait for their episodic care. Studies with more robust and diverse populations are needed to confirm these findings and to implement more engaging models of behavior change.

## Introduction

Preventive measures are critical in avoiding and limiting the severity of diseases. It is intuitive and widely accepted that preventing disease is much more potent than treating morbidities once they have developed. It has been estimated that health behaviors contribute as much as four-fifths of health outcomes, with clinical care only contributing a small proportion of influence [1,2]. This implies that the clinical encounter should always promote behavior changes, emphasizing prevention. However, contemporary healthcare, including emergency medicine, is characterized by scheduling efficiencies, emphasizing shorter times spent in direct contact between the provider and the patient. Direct observations of primary care practices have noted that prevention is also under-emphasized in that setting [3]. Various reasons have been given for the lack of lifestyle change counseling, such as providers not having sufficient time to discuss such matters. Several studies have noted that primary care practitioners spend half or more of their time on activities that are not patient-facing, such as electronic health records (EHRs), insurance, and other tasks. It is thus implicit that new methods are needed to educate patients about self-care and prevention that require less of a clinician’s time. Patients in the emergency department (ED) spend much time waiting for essential processes to occur. In a study that reviewed data from four EDs, waiting times ranged from 15 to 44 minutes, and treatment times ranged from 109 to 177 minutes, the latter of which included waiting for the results of tests, imaging studies, and other activities required for diagnosis and initial treatment [4]. This same study noted that length of stay is essential to patient satisfaction. Despite these long dwell times, ED physicians move rapidly and, therefore, find it challenging to educate patients on the importance of prevention. One study described the median time spent conversing with healthcare providers in the ED as 19 minutes, and that 75% of a patient’s visit was spent not interacting with providers [5]. It is also theorized that educating patients in the ED would likely result in improved health and fewer ED return visits [6]. One study reported that approximately 8.9% of patients return to the same emergency center within 72 hours of leaving it, and 16% within nine days [7]. Patients most commonly return to the ED with concerns about aspects of their original presenting complaints [8]. These statistics show the need to implement disease prevention. The most likely problem with health prevention education is that individuals consider any adverse health outcomes as being offset too far into the future to be of pressing relevance. The ED is likely a place where even chronic and potential future health issues are at the forefront of a person's mind. Hence, they may be more receptive to the importance of lifestyle prevention [6]. ED waiting time may provide an opportunity to educate patients about positive, practical preventive health practices. In addition, the ED visit is often an anxiety-filled experience, with the patient concerned about a potentially severe, life-threatening, or life-changing diagnosis. This may provide a “teachable moment,” wherein a brief preventive intervention may be more effective than one delivered in lower acuity settings.

Receptivity to health behavior changes has been extensively investigated. The transtheoretical model (TTM) is a highly used measurement of receptivity to change and may be used to better understand how to intervene [9]. This model relates that there are six stages people fall into when adopting a more healthful lifestyle. The first stage is precontemplation, in which people have no plans to take action to change their behavior in the near future. This may be due to a lack of information or previous unsuccessful attempts to change. Contemplation occurs when people want to change their behavior within the next six months. They may be more aware of the benefits and costs, but this awareness can create trepidation and cause people to remain in this stage for extended periods of time. The third stage is preparation, which is defined as an individual’s readiness in changing their behavior within the next month. They have a plan and are ready for an action-oriented program. The following stages include action, when changes are initiated within the last six months; maintenance, when people continue positive behaviors and work to prevent relapse; and finally, termination, when people have no temptation to return to their old behavior. The TTM is often applied to preventive behavioral change. For example, it was reported that current smokers typically fall into a spread of 40% precontemplation, 40% contemplation, and 20% preparation [9]. These researchers suggest that when faced with the potential for serious adverse health outcomes, patients may be able to accelerate through some early stages, such as moving from precontemplation to contemplation, or onto the preparation stage.

There have been previous efforts to implement prevention and education in the ED. These attempts include written forms of communication and videos to be used after the clinical encounter as discharge instructions and aftercare instructions, or “after-visit summaries” (AVS), which are paper or electronic documents given to the patient after being seen in the ED to summarize the visit and guide patient health management. However, it has been reported that the AVS is not the most effective tool, considering that only 82.8% of patients recalled receiving an AVS, and 67.4% consulted their AVS [10]. The information provided in the AVS is typically very specific to the reason for that visit and includes contact information for a follow-up appointment. Another study reported an even lower rate, revealing that 51.2% did not recall receiving any AVS [11]. Those who recalled receiving one had lower medication recall scores (remembering the name or color of the bottle or cap, treatment eye(s), and dosing regimen for glaucoma treatment) than those who did not. These results show that the intended goal of the AVS, which is to enhance patient care, was not consistently achieved.

There have also been previous attempts at the prevention of smoking and alcohol use in the ED, using a brief motivational intervention [12,13]. These motivational interventions consisted of 20-30-minute live counseling discussions about the pros and cons of alcohol in the present and the future [14]. The patient was then asked how willing they were to make a change in their drinking habits and related behaviors. The answers were then applied to a readiness ruler that indicated how ready they were to attempt to change their drinking habits and was depicted graphically [14]. Support and follow-up were provided upon completion of the evaluation. In this currently proposed study, patients will be evaluated on how likely they are to attempt to change their lifestyle behaviors and be placed at a stage of the TTM.

Bridging the gap between knowledge and action in preventive health requires exploring new approaches. A promising solution lies in leveraging the waiting time in the ED by offering engaging and easily digestible health information through video education. This readily available resource has the potential to empower patients to make informed decisions and take charge of their well-being. A study involving patients waiting in the ED showed a significant improvement in patient satisfaction after watching a video about what to expect during their visit [15]. The study showed a positive association between watching an educational video and patient satisfaction. Among those who watched the video, 65% rated their ED stay as “excellent” or “very good,” compared to 58.1% of patients who did not watch the video [15]. Patients who received video after-visit guidance scored better on a post-test survey than a standard discharge instructions group in another study [16]. Another smaller study showed that, after viewing an educational video on myocardial infarction, participants in the group shown the video had a statistically significant improvement in their knowledge assessment compared to the group not shown the video [17]. The intervention group had a mean number of correct responses of 27 out of 37 (73%) questions, as opposed to 20 (54%) in the control [17]. These studies may point to the potential value of a video format as a practical and effective approach for promoting lifestyle preventive education.

This project evaluated the impact of a video-based educational intervention administered in the ED following the practitioner’s first encounter with the patient. It examined the feasibility and efficiency of introducing the patient and accompanying guests to the potential benefits that may be derived from adopting essential lifestyle changes. The focused video educated patients on four of the core “pillars of preventive lifestyle” health promulgated by the American College of Lifestyle Medicine, including successful strategies for a plant-based diet, exercise, sleep hygiene, and smoking cessation [18].

## Materials and methods

The Institutional Review Board approved the study as exempt under the implementation of the Final Revised Common Rule in 2017. A waiver of the Health Insurance Portability and Accountability Act (HIPAA) was requested and granted for ED screening. The research team screened adult patients (18-80 years old) admitted to the ED for eligibility based on the inclusion/exclusion criteria (Table 1). The Emergency Severity Index (ESI) is the most commonly used triage system in the United States [19]. The ESI uses a scale from 1 to 5, with one being the highest severity (emergent resuscitation). It should be noted that an ESI of 2 can include normal or abnormal vital signs. In this study, we selected only those patients with an ESI of 2 with normal vital signs, or ESI triage level 3 through level 5, to include lower-acuity patients.

**Table 1:** Eligibility criteria for the study ESI, Emergency severity index

Inclusion criteria	Exclusion criteria
Present with a chief complaint of chest pain	ESI of 1
Adults of ages 18 to 80	Abnormal vital signs
ESI of 3-5, or ESI of 2 with normal vital signs	Non-English speaking patient
-	Unable or unwilling to consent for the study
-	Unable or unwilling to hear a video on a smartphone or computer tablet
-	Patient in infectious disease isolation
-	The patient is over 65 and has an abnormal or positive Orientation-Memory-Concentration Test (OMCT) score
-	The patient is in hospice care

Informed consent, video randomization, and questionnaires were conducted after the physician or advanced practice provider completed the preliminary encounter with the patient, while they were waiting for their results. The video took 10 minutes to view, and the responses to the survey component took an additional 10-12 minutes.

After performing informed consent, the Research Electronic Data Capture (REDCap) [20,21] randomization module assigned patients to the video or control group. All patients then completed a composite questionnaire. The questions were administered orally, or self-administered for those willing to participate, using the tablet interface.

The questionnaire consisted of the validated Readiness-to-Change and Confidence-to-Change questionnaires [22]. Patient confidence in implementing the same behaviors is recorded on a three-point scale. The scores and their descriptions are listed in Table 2.

**Table 2:** Scores for the Readiness-to-Change questionnaire

Score on Readiness-to-Change questionnaire	Description
5 (precontemplation stage)	No interest in the suggested lifestyle change
4 (contemplation stage)	Interest in implementing the lifestyle change in the next few months
3 (preparation stage)	Plan to implement the lifestyle change within the next month
2 (action stage)	Already started the lifestyle change within the past few months
1 (maintenance stage)	Maintained the lifestyle behavior change for over 6 months

The Confidence-to-Change questionnaire addresses barriers or hesitancy that may impact patient confidence in implementing better lifestyle behaviors. The Readiness-to-Change questionnaire aimed to determine which stage of the TTM the patient was in and which behaviors clinicians should focus on first, based on patient-reported readiness. Together, these two questionnaires allow patients and clinicians to develop realistic goals, discuss solutions to barriers, and strategize methods of maintaining better lifestyle behaviors. In addition, the questionnaire included select questions taken from the HCAHPS (Hospital Consumer Assessment of Healthcare Providers and Systems) survey, pertaining to patient satisfaction with their practitioner and visit [23]. 

Several variables, including age, race, ethnicity, sex, gender, and BMI, were obtained from the EHR of those who provided informed consent. Scores were combined to calculate Readiness-to-Change and Confidence-to-Change. These summative scores were used for statistical comparisons. A lower score indicates greater Readiness-to-Change and Confidence-to-Change, respectively.

**Figure 1:**
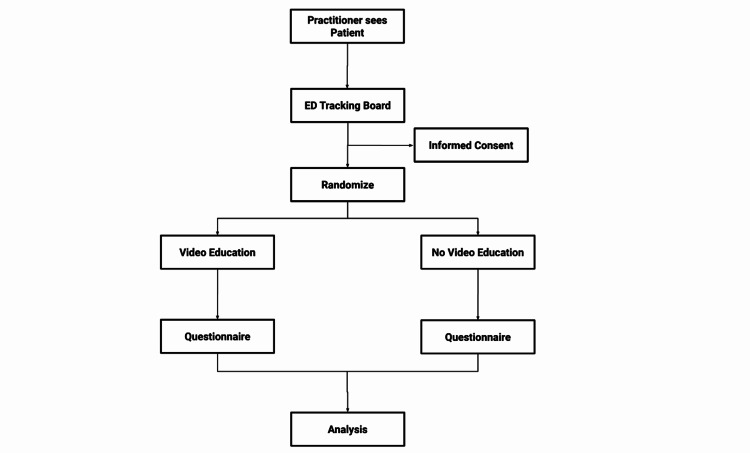
Flowchart for the study process

Research team members were trained to identify the inclusion and exclusion criteria, and use the EPIC emergency medicine tracking board module (Epic Systems Corporation, Verona, WI, USA) to identify candidates for the study [24]. Figure 1 depicts a flowchart of the study process.

## Results

There were 105 subjects enrolled in the study: 60% female, 40% male, and none identified as nonbinary. The mean age of the participants was 48.96 years and a standard deviation of 16.42. The socio-demographic characteristics of the participants are shown below in Table 3.

**Table 3:** Socio-demographic characteristics of the study population

Variables		N	%
Gender	Male	42	40.0
	Female	63	60.0
	Nonbinary	0	0
Race	White participants	69	65.7
	Black/African American participants	32	30.5
	Others	3	2.9
	Unknown	1	1.0
Ethnicity	Hispanic/Latino participants	3	2.9
	Not Hispanic/Latino participants	102	97.1

Table 4 displays the number of participants who were placed into the two cohorts with respect to demographic characteristics.

**Table 4:** Number of participants in each cohort with respect to different characteristics

Variable	Watched video	
	No, n (%)	Yes, n (%)
Overall	61 (59.2)	42 (40.8)
Gender		
Male	19 (47.5)	21 (52.5)
Female	42 (66.7)	21 (33.3)
Race		
White participants	38 (56.7)	29 (43.3)
Black/African American participants	19 (59.4)	13 (40.6)
Others	3 (100)	0 (0)
Unknown	1 (100)	0 (0)
Ethnicity		
Hispanic/Latino participants	3 (100)	0 (0)
Not Hispanic/Latino participants	58 (58)	42 (42)

In this pilot study, the overall mean Readiness-to-Change value was 16.73 for those who watched the video and 17 for those who did not. This indicates that there was improved Readiness-to-Change for those who watched the video. Several findings emerged from the logistic regression model assessing the relationship between various factors and participants' readiness in changing their lifestyle behaviors. Gender did not demonstrate a significant association, with females having an OR of 0.87 (95% CI: 0.39, 1.94; p = 0.738) compared to males. Ethnicity did not exhibit statistically significant associations with Readiness-to-Change value.

Table 5 compares Readiness-to-Change and Confidence-to-Change scores across different age groups. An independent sample t-test was conducted to assess potential age-related variations in these two variables. For Readiness-to-Change, individuals in the 18-39 age group demonstrated a slightly higher mean readiness score (17.71) than those in the 40-80 age group (16.03), implying less Readiness-to-Change, which did not reach significance (p = 0.08). Similarly, the younger 18-39 cohort had a higher summative value for Confidence-to-Change. The 18-39 age group exhibited a higher mean score (23.88) than the 40 to 80 age group (22.39) (p = 0.15, non-significant).

**Table 5:** Independent sample t-test for comparing Readiness-to-Change and Confidence-to-Change scores in different age groups LCL, Lower confidence level; UCL, Upper confidence level

Group	n	Mean	LCL	UCL	p-value
Readiness-to-Change					
Age group					
18 to 39 years	34	17.71	16.04	19.37	0.08
40 to 80 years	71	16.03	14.58	17.48	
Confidence-to-Change					
Age group					
18 to 39 years	34	23.88	22.07	25.7	0.15
40 to 80 years	71	22.39	20.59	24.2	

Although the observed differences did not achieve conventional significance levels, the data shown in Table 6 suggest a potential association between watching the video and increased satisfaction.

**Table 6:** Patients’ satisfaction concerning whether the video was viewed ED, Emergency department

Variable	Watched video		p-value
	No	Yes	
Satisfied with physicians’ courtesy and respect			
No	2 (5.2%)	3 (5.1%)	0.665
Yes	37 (94.8%)	56 (94.9%)	
Satisfied with physicians’ careful listening			
No	3 (7.9%)	6 (10.2%)	0.502
Yes	35 (92.1%)	53 (89.8%)	
Satisfied with physicians’ explanation			
No	6 (42.86%)	8 (13.8%)	0.504
Yes	32 (84%)	50 (86.2%)	
Satisfaction with their ED visit			
No	21 (54%)	28 (48%)	0.536
Yes	18 (46%)	31 (52%)	

Table 7 compares various demographic and health-related variables, such as gender, race, and ethnicity, evaluating the different mean Readiness-to-Change and Confidence-to-Change values observed between individuals who watched the video intervention and those who did not. Among females, there was a slight decrease in mean Readiness-to-Change scores for those who watched the video (16.10) compared to those who did not (17.12), indicating that females were more ready to change when watching the video. Across all racial and ethnic categories, there were improved Readiness-to-Change scores among individuals who watched the video. Shifting to Confidence-to-Change, males who watched the video exhibited a mean Confidence-to-Change score of 23.43, while non-viewers had a slightly higher mean score of 24.68. However, this difference was not statistically significant (p = 0.784). It is noted that, among African American/Black participants, while Readiness-to-Change improved slightly with the video intervention, Confidence-to-Change mildly decreased after watching the educational video.

**Table 7:** Compared Readiness-to-Change scores, with respect to whether the video was viewed, for demographic and health-related variables Note that lower values suggest greater Readiness-to-Change

Variable	Watched video		
	No	Yes	p-value
Readiness-to-Change			
Gender			
Male	16.73 (14.67, 18,81)	17.38 (14.70, 20.06)	0.349
Female	17.12 (15.33, 18.90)	16.10 (13.85, 18.33)	0.757
Race			
White participants	16.57 (14.97, 18.18)	16.52 (14.25, 18.79)	0.519
Black/African American participants	17.53 (14.51, 20.54)	17.23 (14.80, 19.66)	0.559
Others	16.67	-	-
Unknown	24	-	-
Ethnicity			
Hispanic/Latino participants	17.67	-	-
Not Hispanic/Latino participants	16.96 (15.54, 18.39)	16.74 (15.06, 18.42)	0.582
Confidence-to-Change			
Gender			
Male	24.68 (23.11, 26.26)	23.43 (20.62, 26.23)	0.784
Female	23.02 (21.06, 24.99)	22.57 (18.90, 26.25)	0.60
Race			
White participants	23.66 (21.88, 25.43)	21.93 (18.86, 25.00)	0.851
Black/African American participants	22.63 (19.64, 25.63)	25.38 (23.34, 27.43)	0.078
Others	28.33	-	-
Unknown	22.00	-	-
Ethnicity			
Hispanic/Latino participants	26.00	-	-
Not Hispanic/Latino participants	23.41 (21.93, 24.90)	23.00 (20.79, 24.48)	0.627

## Discussion

This exploratory pilot study was conducted to establish the feasibility of a lifestyle-preventive educational intervention in the ED setting, evaluate its impact, and identify the audiences most receptive to adopting healthy lifestyle behaviors.

The time constraints of busy practices are another obstacle to preventive education during the patient encounter. A typical emergency practitioner cannot provide meaningful face-to-face education on lifestyle behaviors in the context of a busy practice. The current pilot study addresses this issue with a video educational intervention that minimally impacts the clinician’s workflow.

If an institution experiences improved patient satisfaction with the practitioner or host institution, this may motivate organized health systems to adopt this preventive education as a regular practice. In our study, patients showed a trend toward increased satisfaction with their hospital visits and providers. However, the sample size in this pilot study was not adequate to establish significance. Subjects were aware that this was a research project, and the speaker in the video was not usually their provider in the ED. Therefore, they may not have associated being shown an educational video on prevention with their provider taking the time to ensure they live healthier. Most importantly, this pilot study demonstrates the feasibility of using video education in the ED. The implication is that the emergency setting can be a “teachable moment” for preventive education.

Another consideration that may be raised is the idea of provider cynicism or nihilism. The ED presents a unique opportunity to educate patients and address provider cynicism. Healthcare cynicism has been defined as the decline in empathy and emotional neutralization that most likely begins during medical training, “from student to physician” [25]. Nihilism, in this context, can be considered the tendency of practitioners to think that educating patients on the importance of lifestyle preventive behaviors is futile. Provider cynicism or nihilism towards lifestyle prevention can significantly hinder the integration of these strategies into patient care. Such attitudes contradict the role of healthcare practitioners as educators and coaches, ultimately impacting the promotion of preventive health.

An explorative study in Austria surveyed 99 medical students three times across their training [26]. Their responses demonstrated increased cynicism throughout their medical school journey, from first to third years. Those who continuously expressed high cynicism reported lower psychological well-being and decreased optimism [26]. Another interview study of medical students in the ED found a recurring theme of cynicism and a trend of decreased empathy, which became more pronounced as they progressed from medical students to attending physicians [27]. This study also associated cynicism with burnout indices [27]. Being empathetic and compassionate when dealing with patients is crucial in improving their well-being and health outcomes [27]. The use of a preventive educational video that minimizes interference with the practitioner's workflow may decrease cynicism because it does not require direct provider education.

It is essential to discuss this study’s strengths and weaknesses. The study was conducted at one ED in a suburban teaching hospital, which presents limitations for the generalizability of the results. A more extensive, multicenter study that includes different populations should be conducted to address this issue. Also, the current study utilized a passive video instruction tool. An interactive video approach may be a more impactful and effective educational method for future studies. It has been reported that an interactive educational video approach is more effective than a traditional online classroom, as measured by mean test scores [28].

## Conclusions

This pilot study explored the feasibility of a randomized preventive video education intervention in the ED. The ED offers a unique “teachable moment,” when patients are more receptive to lifestyle changes due to heightened health awareness. While this exploratory study demonstrated feasibility and trends toward increased Readiness-to-Change scores following the video intervention, a larger study is needed to confirm these findings. Future research may also benefit from a more interactive video format.
